# Tracking sub-syllabic features in zebra finch song during development

**DOI:** 10.1186/1471-2202-13-S1-P20

**Published:** 2012-07-16

**Authors:** Meagan E Woodford, Matthew M Benavides, Todd W Troyer

**Affiliations:** 1 Biology Department, Neurosciences Institute, UTSA, San Antonio, Texas, 78249, USA

## 

Most natural behaviors are arranged hierarchically, with complex actions composed of serial combinations of more basic motor gestures. The learning of courtship song in birds presents an ideal model system for understanding the neural mechanisms underlying such learning. In the most commonly studied species, the zebra finch, songs are learned by young males from their fathers or other male tutors. They are highly stereotyped by adulthood, and yet vary greatly between individual birds. The basic element of song is generally considered to be the syllable, a period of continuous expiratory vocalization separated by silence and a brief period of inspiration. These brief silences make it relatively easy to segment the acoustic signal into syllables, and previous studies have examined song development by extracting acoustic features from the sound spectrogram and then averaging these features over syllables [1,2]. However, despite experimental evidence that syllables form behavioral units, the syllabic structure of song is not readily apparent in many electrophysiological recordings. Individual neurons in the song motor pathway generally produce 5-10 msec bursts of activity that are aligned to song production with millisecond precision and have no obvious relationship to syllable onsets or offsets.

We have developed a suite of software that enables semi-automatic the tracking of sub-syllabic features in a large corpus of acoustic data recorded from developing zebra finches with live tutors. Our approach is based on three basic ideas. First, we segment song into syllables and annotate individual syllables based on averaged feature values in a manner similar to previous approaches. Second, we use a coarse temporal alignment to average across renditions and produce an averaged ‘template’ for each syllable type. Then we use dynamic time warping to align each syllable rendition to this averaged template. Finally, we measure how well each rendition matches the template, and use this measure to perform semi-automated screening of possible outliers and syllable misclassifications.

We are currently applying this approach to track the development of song features during the later stages of song development. To quantify the degree to which the mechanisms of song development respect a division of song into syllable-based units, we will measure song-by-song correlations between different features in different sub-components of the same syllable and compare these to feature correlations across syllables.

